# A Bayesian approach to estimating hidden variables as well as missing and wrong molecular interactions in ordinary differential equation-based mathematical models

**DOI:** 10.1098/rsif.2017.0332

**Published:** 2017-06-14

**Authors:** Benjamin Engelhardt, Maik Kschischo, Holger Fröhlich

**Affiliations:** 1 Rheinische Friedrich-Wilhelms-Universität Bonn, Algorithmic Bioinformatics, Bonn, Germany; 2 DFG Research Training Group 1873, Rheinische Friedrich-Wilhelms-Universität Bonn, Germany; 3 Department of Mathematics and Technology, University of Applied Sciences Koblenz, RheinAhrCampus, Remagen, Germany; 4 UCB Biosciences GmbH, Monheim, Germany

**Keywords:** ordinary differential equations, systems biology, dynamic elastic-net, modelling

## Abstract

Ordinary differential equations (ODEs) are a popular approach to quantitatively model molecular networks based on biological knowledge. However, such knowledge is typically restricted. Wrongly modelled biological mechanisms as well as relevant external influence factors that are not included into the model are likely to manifest in major discrepancies between model predictions and experimental data. Finding the exact reasons for such observed discrepancies can be quite challenging in practice. In order to address this issue, we suggest a Bayesian approach to estimate hidden influences in ODE-based models. The method can distinguish between exogenous and endogenous hidden influences. Thus, we can detect wrongly specified as well as missed molecular interactions in the model. We demonstrate the performance of our Bayesian dynamic elastic-net with several ordinary differential equation models from the literature, such as human JAK–STAT signalling, information processing at the erythropoietin receptor, isomerization of liquid *α*-Pinene, G protein cycling in yeast and UV-B triggered signalling in plants. Moreover, we investigate a set of commonly known network motifs and a gene-regulatory network. Altogether our method supports the modeller in an algorithmic manner to identify possible sources of errors in ODE-based models on the basis of experimental data.

## 1. Introduction

Mathematical models of biological systems become more and more complex and contribute important insights into various biological processes [1–7]. Since biological systems are naturally open, formulating mathematical models and specifying their boundaries is a highly non-trivial task [8,9]. Consequently, most researchers in systems biology are faced with the still unsolved issue to find a compromise between model complexity and the limited amount of knowledge, data and time [9–11]. Researchers in other fields including earth and environmental sciences are facing similar challenges [12]. In many cases, missed and unknown external influences as well as erroneous interactions in a model could lead to severely misleading results [7].

Current research is mostly focused on inference of perturbation effects and model selection [13–15]. Although, perturbation experiments are labour and cost intensive, which raises the need for a careful prioritization strategy [14–17]. On the other hand, statistical model selection and related methods require a strong knowledge about the system and its alternatives which is rarely given in practice [7,18]. Thus model selection can be very difficult, specifically if nothing is known about missing variables and their possible mechanisms.

In most situations, researchers have partial knowledge and preliminary hypotheses about their system, which needs to be integrated into a restricted but still predictive and experimentally validatable model [19]. Even if the biological system is partially known and the data are given for almost all molecular species, it is not clear how to deal with insufficient predictions [7]. Often this ends in trial-and-error approaches and does not ensure that the selected model reflects the reality rather than just fitting the given dataset. The question is, how to detect so far unknown molecules and their interactions in a more data driven manner. This could guide the modeller towards points in the given model, where the model is likely erroneous. In a second step, the modeller can then try to link these erroneous points to known mechanisms.

Recently, we proposed the dynamic elastic-net (DEN) as a more principled method to address this issue [20]. The DEN aims for estimating the dynamics of hidden influence variables in ordinary differential equations (ODE)-based systems via a penalized estimation procedure resembling elastic-net regression [21]. While our previous method was tested successfully on several applications, such as the erythropoietin receptor (EpoR)-dependent signalling network [19], it has still several shortcomings, which we address in this paper. More specifically, DEN is not a probabilistic approach and thus does not fully address the unavoidable uncertainty about estimates. DEN does not answer the question, whether estimated hidden influences could be attributed to missed or wrongly modelled interactions among the known molecular species. Here we introduce the Bayesian DEN (BDEN) as a new and fully probabilistic approach, which deals with all these aspects. In contrast with DEN, our new BDEN method does not require pre-specified hyper-parameters. We illustrate the predictive power of BDEN compared with DEN in several real biological models and test cases. The BDEN thus provides a systematic Bayesian computational method to identify target nodes and reconstruct the corresponding error signal including detection of missing and wrong molecular interactions within the assumed model. The method works for ODE-based systems even with uncertain knowledge and noisy data. In contrast with approaches based on point estimates the Bayesian framework incorporates the given uncertainty and circumvents numerical pitfalls which frequently arise from optimization methods [22,23].

## 2. Material and methods

### 2.1. Motivation

**Figure 1.**
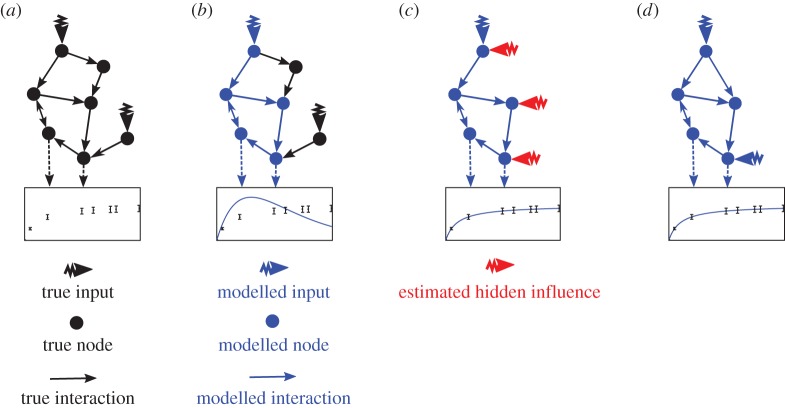
Illustration of the Bayesian approach to estimating hidden influence variables in ODE-based models. Here, a fictional interaction network is shown. (*a*) True system (black) with two inputs and one fictive observable measurement as a combination of two nodes (box). (*b*) In reality, the available knowledge represented by an *a priori* model (blue) does not necessarily cover the whole system but only a part of the true system. Hence the nominal model leads to an unsatisfactory fit with the observable measurement. This may caused by exogenous influences or by misspecified molecular interactions (i.e. missing or wrong edges in the interaction network). (*c*) Our approach aims for estimating these hidden influences (red) and the directly involved molecular species. (*d*) Some of the estimated hidden influences may correspond to missing or wrong molecular interactions *within* the system. Hence, in a last step, our method tries to further distinguish between intrinsic and exogenous hidden influences. We therefore identify erroneous parts of the nominal ODE system and give detailed hints for their correction.

We assume the modelling process to start with an initial, potentially incomplete or partially misspecified nominal model including all known but not necessarily observable molecules [24]. Figure 1 illustrates the general idea. In most situations, the real system differs from the initially modelled nominal system, which is reflected by an insufficient fit to the given data caused by (i) hidden influences and (ii) erroneous molecular interactions. Exogenous hidden influences could, for example, be stimulatory (e.g. phosphorylation) or inhibitory (e.g. de-phosphorylation) events affecting the modelled system from outside. In addition, there could exist stimulatory or inhibitory influences *within* the system, which are not included in the model due to lack of knowledge, i.e. missing molecular interactions. Similarly, wrongly included molecular interactions could exist. In biochemical systems, molecular interactions are based on biochemical reactions, e.g. phosphorylation and binding events.

Owing to the fact that biological systems are open, the number of potential erroneous nodes (e.g. proteins or other molecules) within the nominal model is huge [9]. Without further knowledge, independent error terms have to be assigned to each node. If the respective node is in reality not directly targeted by a hidden influence, the hidden input takes the value zero. Only nodes directly affected by hidden influences have non-zero errors. Wrongly modelled or missing interactions between two nodes can be represented by two error terms, one for each of the respective nodes, which will be correlated over time. We exploited this idea to detect missing or erroneous interactions in a given ODE-based nominal model.

### 2.2. Approach

We assume the dynamical model2.1a
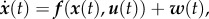
2.1b
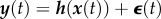
2.1c
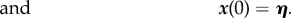
Here, 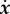 denotes the time derivative of the state vector 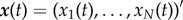 with initial value ***η***. The not necessarily linear function ***f*** represents the nominal model, which describes the current assumptions about the dynamic interactions between the state variables and in addition the effect of the known input function 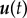.

No model of a biological system can ever be totally complete and comprehensive. Therefore, we add the hidden influences 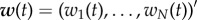 to the nominal model function ***f***. The additive dynamic hidden influence ***w***(*t*) subsumes missing or wrong interactions between the state variables as well as exogenous influences caused by crosstalk with other biological processes (see electronic supplementary material, §3). Of course, ***w***(*t*) is unknown and we aim to estimate these hidden influences from the data [20]. Notably, the hidden influence ***w***(*t*) is not restricted to be constant or linear and thus can be any arbitrary function of time.

Often it is impossible to measure all components of the state vector ***x***, e.g. concentrations of reacting substances (due to technical limitations, e.g. non-availability of phospho-protein specific antibodies). The map from the state to the measurable output 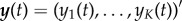, with *K* not necessarily equal to *N*, is given by the measurement function ***h***, which we assume to be known (equation (2.1*b*)). In addition, we assume white Gaussian measurement noise 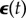 with expectation zero and a noise covariance matrix 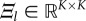, see below.

In practice, the data are given as measurements 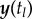 at discrete time points *t*_*l*_ with *l* ∈ {1, …, *T*}. We will use the notation *y*_*k*,*l*_ = *y*_*k*_(*t*_*l*_) for the measured output *k* ∈ {1, …, *K*} at measurement time *t*_*l*_ and the analogous notation for the other variables, i.e. *x*_*i*_(*t*_*l*_) = *x*_*i*,*l*_ and 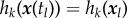. For sake of simplicity in the following, we denote the matrix of observed measurements by 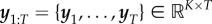 and the corresponding state and hidden influence matrices by 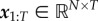 and 
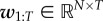
.

From now on, we are interested in hidden influences at discrete time points. Under this assumption equation (2.1) can be rewritten as2.2a
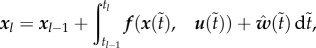
2.2b
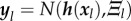
2.2c
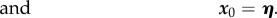
Consequently, we obtain a first-order Markov process over the state variables ***x***. The function 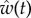 is obtained by fitting a cubic smoothing spline through each of the *N* discrete time series of hidden influence signals *w*_*i*,1:*T*_ [25].

The assumption of Gaussian measurement noise can, if necessary, approximately be fulfilled after a variance-stabilizing transformation [26]. In addition, we assume uncorrelated measurement noise and thus *Ξ*_*l*_ = diag(*ξ*^2^_1,*l*_, …, *ξ*^2^_*K*,*l*_).

### 2.3. Marginal likelihood of the data

The likelihood of the observed data2.3
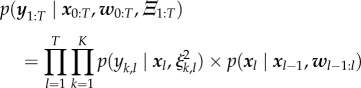
can be factorized due to the independence of the measurement noise with respect to time and observables. Note that 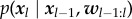 is defined by equation (2.2a). In addition, 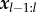 and 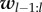 are conditionally independent from *Ξ*_1:*T*_.

Since typically the number of replicate measurements per time point is small, the empirical variance is not a reliable estimator of the true measurement noise. Therefore, we impose an inverse gamma prior on the variance of the measurement noise2.4
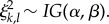
The marginal likelihood of the data are obtained by marginalizing over the variance of the measurement noise variable2.5
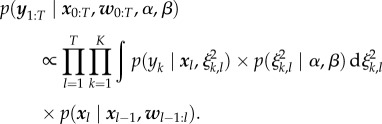
This integral can analytically be calculated to yield [27]2.6
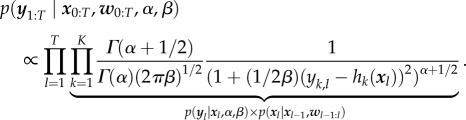
A detailed derivation of equation (2.6) is provided in electronic supplementary material, §4.

According to Bayes' theorem, the posterior density over the hidden input signals 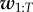 with initial value 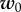 is given by2.7
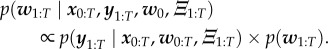
Using equation (2.6), we can directly draw samples from the posterior density of the hidden influence. For this purpose, we propose a Bayesian elastic-net prior as detailed in the following sections.

### 2.4. Smoothness and sparsity via a Bayesian elastic-net prior

**Figure 2.**
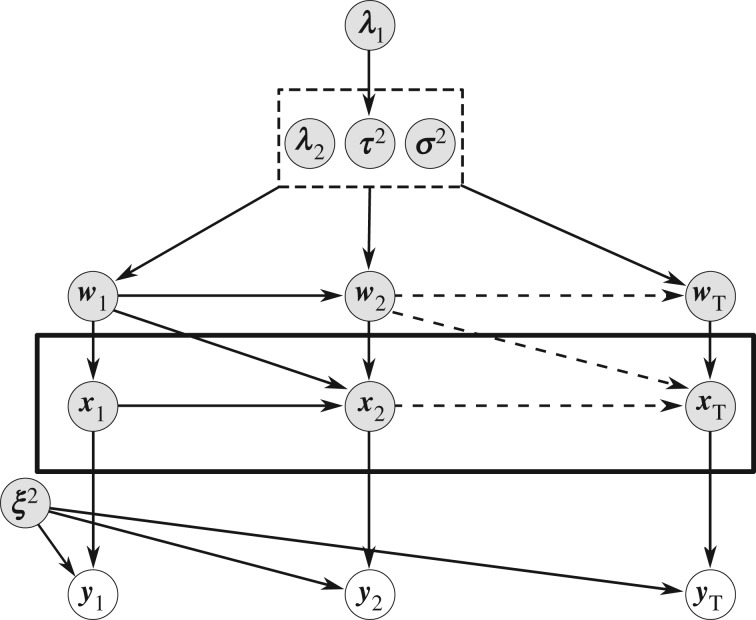
Representation of the proposed Bayesian dynamic elastic-net approach as a probabilistic graphical model. The hidden influences ***w***_l_ form a Markov chain over all time points *l* = 1, …, *T* and are directly dependent on the shared parameters 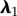 and ***λ***_2_. Since the outcome of one integration step represents the initial value for the next integration step, the system state variables 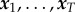 are also successively dependent.

The hidden input signals 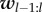 can be understood as the statistical residuals of the nominal system, and every deviation of observations from the nominal system could thus be explained by non-zero components in 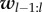. However, we are only interested in hidden input signals, which are far stronger than measurement noise. Therefore, we assume that the hidden input signal is smooth and sparse. Sparsity corresponds to the *a priori* belief that only a small subset of state variables is truly affected by unknown external or internal input signals. In addition, we assume the hidden input signal to be smooth over time. Smoothness and sparsity are encoded by a prior distribution inspired by the Bayesian elastic-net, which is here combined with a first-order Markov process over 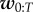 [28,29]. Overall our proposed approach is thus a hierarchical graphical model shown in figure 2. Details can be found in electronic supplementary material, §5.

Briefly, the Bayesian elastic-net defines a conditional Gaussian prior over each *w*_*i*,*l*_|*w*_*i*,*l*−1_ (*i* = 1, …, *N*, *l* = 1, …, *T*). The scale of the variance of the Gaussian prior is a strongly decaying and smooth distribution peaking at zero, which depends on parameters *λ*_2_, 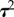 and *σ*^2^. The parameter 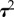 is itself given by an exponential distribution (one for each component of vector 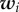) with parameters 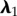. In consequence, sparsity is dependent on the parameter vector 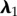, whereas smoothness is mainly controlled by *λ*_2_ [28,30]. These parameters are drawn from hyper-priors, which can be set in a non-informative manner or with respect to prior knowledge about the degree of shrinkage and smoothness of the hidden influences [31]. We refer the reader to electronic supplementary material, §§5 and 7, for details.

### 2.5. Estimating hidden influences from data

To estimate the hidden input and the parameters in the hierarchical model, we devise a Metropolis–Hastings algorithm with Gibbs updates of the Bayesian elastic-net hyper-parameters. The algorithm proceeds sequentially between the different time points 1, …, *T* by drawing different samples at each supporting point. At sampling step *s* and time point *l*, a random component *w*_*i*,*l*_ is selected of the hidden input vector (a node in the network) at the previous time point, which is modified by a sample from a univariate Gaussian transition kernel *π* [32]. The resulting vector 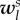 is accepted with probability2.8
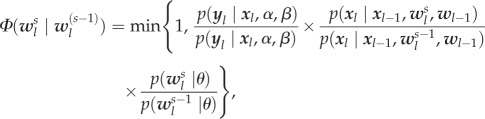
where 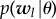 is the Bayesian elastic-net prior over the hidden influences conditioned by hyper-parameters 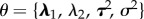 (for details, see electronic supplementary material, §§5 and 7). Because of the Gaussian measurement errors, the discrepancy between data component *y*_*k*,*l*_ and the corresponding model output 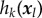 in equation (2.6) is given by the quadratic error 
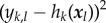
. Note that 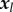 is obtained by numerically integrating the ODE system from time point *l* − 1 using 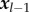 as initial value according to equation (2.2a). Code for the sampling algorithm is provided in electronic supplementary material, §6.

### 2.6. Estimating endogenous hidden influences: missing and wrong reactions

**Figure 3.**
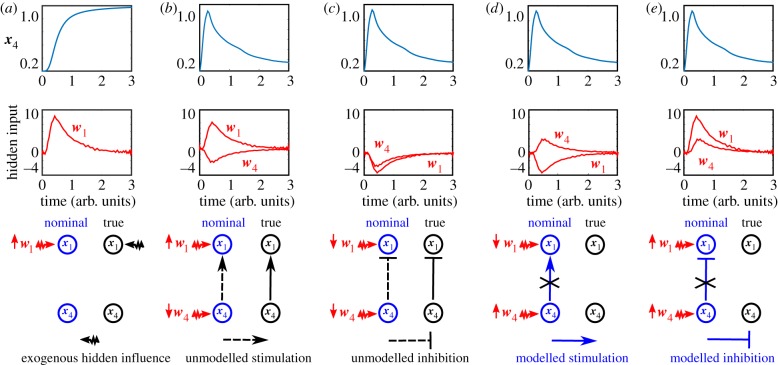
Illustration of hidden exogenous and endogenous influences by an arbitrary example system. (*a*) Hidden exogenous influence. No significant temporal correlation between ***x***_4_ and ***w***_1_ is expected. (*b*) Hidden endogenous influence as a missing stimulatory interaction (arrow) from ***x***_4_ to ***x***_1_. Here, hidden influences ***w***_4_ and ***w***_1_ are highly negatively correlated. This is caused by a missing stimulating effect of ***x***_4_ on ***x***_1_. The decrease of ***x***_4_ is correlated with an increase of ***w***_1_. (*c*) Hidden endogenous influence as a missing inhibitory interaction. Here, hidden influences ***w***_4_ and ***w***_1_ have a strong positive correlation and compensate a missing inhibitory effect of ***x***_4_ on ***x***_1_. The increase of ***x***_4_ is correlated with an decrease of ***w***_1_. (*d*) Hidden endogenous influence as erroneous stimulation. Here, hidden influences ***w***_4_ and ***w***_1_ have a strong negative correlation. The increase of ***x***_4_ goes along with a decrease of ***w***_1_. (*e*) Hidden endogenous influence as an erroneous inhibition. The hidden influences ***w***_1_ and ***w***_4_ are concordant and correlate strongly with the state component ***x***_4_.

After having estimated hidden influences on state components in the nominal ODE system, the question arises whether these hidden variables could in fact correspond to missing or wrongly specified interactions within the nominal system. A simple strategy, which we followed here, is to rank all state variables in the nominal system by their temporal correlation with the estimated hidden influences. The essential idea is that in case of a wrong or missing reaction the estimated hidden time courses should ‘compensate’ erroneous predictions by the nominal system (figure 3). In general, wrong or missing interactions can either have an increasing (stimulatory) or decreasing (inhibitory) influence on the target nodes. This results in a negative hidden influence with 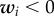 in the case of inhibition and a positive hidden influence with 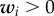 in the case of stimulatory events. More specifically, we distinguish several cases which are listed in table 1 and further illustrated in figure 3. Briefly, the idea is that an modelled stimulation between two state variables ***x***_1_, ***x***_4_ in the ODE system yields two error signals ***w***_1_ (influencing ***x***_1_) and ***w***_4_ (influencing ***x***_4_), which are anticorrelated. This is because ***w***_1_ and ***w***_4_ capture the unmodelled dynamics of the ODE system. Similarly, an unmodelled inhibition yields ***w***_1_, 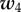 and a positive correlation of ***w***_1_ and ***w***_4_. A wrongly modelled stimulation results in 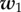 and 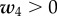 which are anticorrelated. A wrongly modelled inhibition yields ***w***_1_, 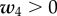 and a positive correlation.

**Table 1.** An endogenous hidden influence is expected to yield different types of (cross-)correlations with other hidden influences (Corr. HI) and state variable dynamics (Corr. State), depending on whether a molecular interaction is missing or wrongly specified in the nominal system. Missing and wrong molecular interactions can be further distinguished depending on whether the true molecular interaction is of stimulatory (stim.) or inhibitory (inh.) nature. Furthermore, molecular interactions between two species can either be modelled in the correlation between the target hidden influence dynamics and the related estimated model variables (Corr. State). In case of endogenous hidden influences, these correlations are expected to be either strongly positive (+) or strongly negative (−) if they reflect true molecular interactions. Figure 3 illustrates all four cases in detail.

	event	Corr. HI	Corr. State
missing	stim.	**−**	**+**
	inh.	**+**	**−**
wrong	stim.	**−**	**−**
	inh.	**+**	**+**

As exemplified in figure 3, due to differences in the expected correlations, our analysis should also allow for distinguishing missing inhibitory versus stimulating effects of missing monotonous interactions.

Different measures exist to capture the strength of correlation between time courses. Apart from the Pearson correlation, we here used the the cross-correlation coefficient2.9
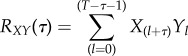
to quantify temporal associations between two time series *X* and *Y* [33]. The cross-correlation *R*_*XY*_(*τ*) depends on the time lag *τ* which was chosen as argmax_*τ*_[*R*_*XY*_ (*τ*)].

## 3. Results

### 3.1. Tested mathematical models

The EpoR-induced JAK–STAT signalling pathway mediates a rapid signal transduction from the receptor to the nucleus related to cell proliferation and differentiation [19]. This pathway involves a rapid nucleocytoplasmic cycling of the signal transducer and activator of transcription 5 (STAT5) molecules which is not directly measurable [19].

The G protein cycling model quantitatively characterizes the heterotrimeric G protein activation and deactivation in yeast [34]. This model serves as a fully observed but complex test case where all states are measured.

In contrast, the model of the UV-B signalling in plants systematically links several signalling events induced by UV-B light to a comprehensive informational signalling pathway [35]. Only combinations of small amounts of the involved molecules are accessible and thus it serves as a complex and not fully observed test case.

Network motifs are thought to represent building blocks of larger biological systems [36]. It is thus informative to test BDEN with respect to these motifs to better understand the possible dependency of the performance of our models on different basic network topologies.

The dynamic EpoR model reflects the information processing at EpoR including turnover, recycling and mobilization of EpoR after stimulation with erythropoietin (Epo) at the cell membrane [37]. Consequently, it details the first part of the JAK–STAT signalling pathway. Only combinations of Epo concentrations in the medium, on the surface and in the cells are accessible and thus it represents a complex model with limited experimental data [37].

By contrast, the thermal isomerization of *α*-Pinene (*α*P) in the liquid phase has the purpose to investigate the applicability of BDEN to small compound reaction networks [38]. The model details the racemization of *α*P and its simultaneous isomerization to dipentene (dP) and allo-ocimene (aO).

To further investigate the utility of BDEN for complex systems, we used a gene-regulatory network composed of six genes and related proteins obtained from the DREAM6 challenge [39]. Further details about the described models are given in electronic supplementary material, §8.

### 3.2. Simulation study: testing the performance of Bayesian DEN

We first compared the performances of BDEN as well as our old DEN approach to correctly predict the location of single hidden influence for the JAK–STAT signalling model, the heterotrimeric G protein cycling, the U-VB signalling in plants and the aforementioned network motifs [19,34–36]. This was done on the basis of simulated data for each system. Details about the simulations are given in electronic supplementary material. For BDEN, we computed for each 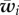 (where 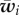 denotes the posterior mean taken over MCMC samples) the area under the predicted hidden influence curve by trapezoidal numerical integration [40]. For DEN, we applied the same method based on the provided point estimates of hidden influence curves. The area under the predicted hidden influence curve was compared against the simulated existence and non-existence of a hidden signal at that node. Consequently, we were able to compute an area under ROC (AUC) value and a corresponding Brier score (BS), i.e. the squared difference between the prediction score and the Boolean indicator of a true hidden influence [41,42]. Table 2 and electronic supplementary material, table S1, show a favourable overall performance of our new method for different levels of measurement noise and simulated errors of kinetic parameter estimates.

**Table 2.** Performance of BDEN and DEN regarding the dependence on measurement noise (median). The median absolute deviation for the AUC (ROC) and Brier score (BS) are given in brackets.

model	noise level	method	AUC	BS
JAK–STAT	2.5%	BDEN	0.90 (0.15)	0.11 (0.11)
		DEN	0.60 (0.40)	0.16 (0.06)
	7.5%	BDEN	0.83 (0.18)	0.21 (0.19)
		DEN	0.43 (0.29)	0.30 (0.14)
	12.5%	BDEN	0.75 (0.25)	0.26 (0.16)
		DEN	0.42 (0.31)	0.41 (0.12)
G protein	2.5%	BDEN	0.99 (0.02)	0.04 (0.03)
		DEN	1.00 (0.00)	0.09 (0.02)
	7.5%	BDEN	0.88 (0.13)	0.17 (0.09)
		DEN	0.80 (0.13)	0.16 (0.09)
	12.5%	BDEN	0.80 (0.16)	0.22 (0.10)
		DEN	0.71 (0.16)	0.20 (0.11)
UV-B	2.5%	BDEN	0.91 (0.11)	0.19 (0.06)
		DEN	0.80 (0.19)	0.22 (0.08)
	7.5%	BDEN	0.88 (0.14)	0.19 (0.04)
		DEN	0.80 (0.19)	0.20 (0.06)
	12.5%	BDEN	0.81 (0.15)	0.19 (0.05)
		DEN	0.71 (0.15)	0.19 (0.05)
motifs	2.5%	BDEN	1.00 (0.00)	0.00 (0.00)
		DEN	0.90 (0.14)	0.11 (0.09)
	7.5%	BDEN	1.00 (0.00)	0.00 (0.00)
		DEN	0.81 (0.19)	0.19 (0.04)
	12.5%	BDEN	1.00 (0.00)	0.00 (0.00)
		DEN	0.80 (0.15)	0.19 (0.05)

Next, we investigated the performance of BDEN to correctly detect more than one hidden influence. We used the comparatively large G protein cycle model for this purpose. Results can be found in electronic supplementary material, table S2. In this simulation, we randomly added hidden influences for up to 50% of the nodes and still observed a good prediction performance.

In a similar manner, we investigated the performance of BDEN to detect wrong and missing interactions (table 3). We simulated wrong model specifications of the heterotrimeric G protein cycling, the UV-B signalling in plants and the synthetic JAK–STAT signalling by randomly removing and adding interactions. As described above, the quantitative predictions of BDEN are given in terms of (cross-)correlations. By comparing these correlation values against the true existence and non-existence of a particular interaction, we were able to compute an AUC value. Notably, missing and wrong interaction detection is only possible with our new BDEN approach. Again we archived a very good performance for all systems under investigation. On average, 80% of the missing interactions are correctly detected by BDEN. Among the correctly identified missing interactions, on average 90% were correctly classified as ‘stimulating’ and ‘inhibiting’, respectively (electronic supplementary material, table S3). Details regarding the dependency on the measurement noise are given in table 3 and results in dependency of deviance of the parameter estimates are given in electronic supplementary material, tables S4 and S5.

**Table 3.** Performance of BDEN to detect wrong and missing interactions depending on the measurement noise (median) evaluated for the JAK–STAT (JS), G protein (GP) and UV-B network. The median absolute deviation for the AUC (ROC) is given in brackets.

	model	noise level	AUC
missing interaction	JS	2.5%	1.00 (0.00)
		7.5%	0.83 (0.28)
		12.5%	0.80 (0.32)
	GP	2.5%	0.81 (0.19)
		7.5%	0.78 (0.22)
		12.5%	0.62 (0.47)
	UV-B	2.5%	1.00 (0.00)
		7.5%	0.91 (0.11)
		12.5%	0.76 (0.16)
wrong interaction	JS	2.5%	1.00 (0.00)
		7.5%	0.87 (0.32)
		12.5%	0.80 (0.23)
	GP	2.5%	1.00 (0.00)
		7.5%	0.81 (0.32)
		12.5%	0.73 (0.40)
	UV-B	2.5%	0.81 (0.20)
		7.5%	0.70 (0.24)
		12.5%	0.68 (0.30)

### 3.3. Examples with real data

In the following, we further illustrate the results obtained with BDEN for the JAK–STAT signalling model, the information processing at EpoR and the isomerization of *α*P using experimental data.

#### 3.3.1. JAK–STAT signalling

The JAK–STAT signalling pathway model (§3.1) consists of four molecular species.

Unbound STAT5 molecules become phosphorylated (STAT5_p_) catalysed by the erythropoietin receptor. Two STAT5_p_ molecules can form a dimer (STAT5_di_) and thus are able to enter the nucleus (STAT5_n_). Only the amount of phosphorylated STAT5 molecules, the total amount of STAT5 and the erythropoietin receptor are directly accessible. Experimental measurements are available at 16 time points [19].

**Figure 4.**
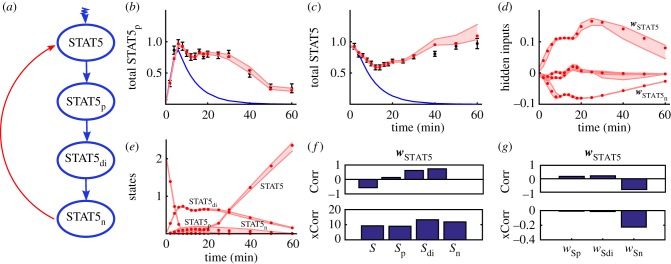
Reconstructing the model error for the JAK–STAT signalling pathway [19]. (*a*) Nominal model of the JAK–STAT signalling (blue) including its nucleocytoplasmic cycling (red). (*b*,*c*) Measurements (black) for phosphorylated cytoplasmic STAT5 and total cytoplasmic STAT5 and posterior BDEN predictions including the 95% credible intervals (red) based on the nominal system (blue). (*d*) Estimated hidden inputs (posterior means) and 95% credible intervals. There is a clear input located at STAT5 and STAT5 in the nucleus. (*e*) Estimated time series posterior means for all modelled variables including 95% credible intervals. (*f*) Estimated correlations (Corr) and cross-correlations (xCorr) of the estimated hidden input 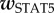 located at STAT5 with all estimated state variables. (*g*) Estimated correlations and cross-correlations of 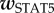 with all remaining hidden influences. Here 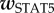 is clearly correlated to the hidden input located at STAT5_n_ and in addition it is correlated to the time series of STAT5_n_. High cross-correlation is a necessary but not sufficient condition.

Figure 4 illustrates the application of our method when ignoring the back-translocation of STAT5_n_ into the cytoplasm, which was hypothesized by the authors [19]. After parameter fitting the nominal system is not in sufficient agreement with the data. Introducing hidden influence terms 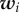 leads to good agreement with the observations. Our method clearly localized two hidden influences 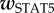 and 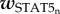 at STAT5 and STAT5_n_. Subsequent analysis shows a high positive (cross-)correlation of 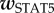 with STAT5_n_ and a negative one with 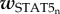. Exactly the opposite pattern can be observed for 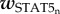. According to our above explained procedure we thus predict a stimulatory influence of 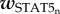 on STAT5. This is in agreement with the claimed nucleocytoplasmic cycling of the phosphorylated STAT5 dimer [19,43].

#### 3.3.2. EpoR model

The complex core model of the EpoR regulation via receptor mobilization, turnover and recycling involves six species and eight time points. Here the ligand Epo binds to EpoR on the surface and builds a ligand–receptor complex (Epo–EpoR). In consequence, Epo–EpoR triggers the phoshorylation of the cytoplasmic EpoR and thus induces the JAK–STAT signalling pathway [37]. Several mechanisms affect the amount of active EpoR. This model covers the ligand-induced receptor endocytosis and thus the internalization of the ligand-bound receptor (Epo–EpoR_*i*_), receptor recycling and degradation of the internalized ligand-bound receptor. Location-dependent degradation results in degraded Epo in cytoplasm (dEpo_*i*_) and in medium (dEpo_*e*_).

**Figure 5.**
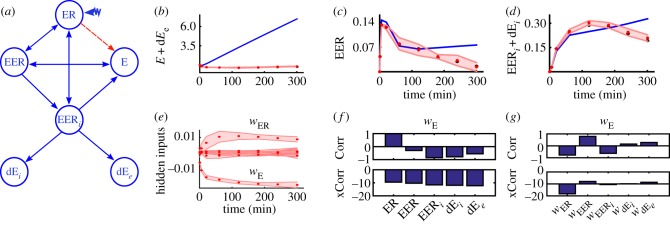
Reconstructing the hidden influence in a model of the EpoR regulation [37]. (*a*) Reaction graph of the model (E = Epo, ER = EpoR, EER = Epo − EpoR, EER_*i*_ = Epo − EpoR_*i*_, dE_*i*_ = dEpo_*i*_, dE_*e*_ = dEpo_*e*_). The red arrow is a wrong reaction. (*b*,*c*,*d*) Output measurements (black) compared with posterior BDEN predictions (red) including 95% credible intervals and the nominal model (blue). (*e*) Estimates of the hidden influences (posterior mean) including 95% credible intervals. (*f*) Estimated correlations (Corr) and cross-correlations (xCorr) of the hidden influence related to *w*_E_ with all estimated state variables. A clear correlation with EpoR is observable. (*g*) Estimated correlations (Corr) and cross-correlations (xCorr) of the hidden influence related to *w*_E_ with all estimated remaining hidden influences. Again *w*_E_ is clearly correlated with *w*_ER_. In consequence, the direct interaction between ER and E is correctly classified as wrong and has to be removed.

As a test case for BDEN, we wrongly specified a receptor-induced feedback on the amount of available Epo. In consequence, we expect to detect this wrong interaction. As shown in figure 5, BDEN allows to correctly localize and characterize this erroneous interaction in the nominal model.

#### 3.3.3. *α*P isomerization

The model of the dynamic isomerization of *α*P is composed of four molecular species. Measurements of *α*P, dP, aO and the dimer (Di) are available [38]. After heating, *α*P reacts either to dP or builds a dimer by reacting with aO. Furthermore, Di can react to aO.

**Figure 6.**
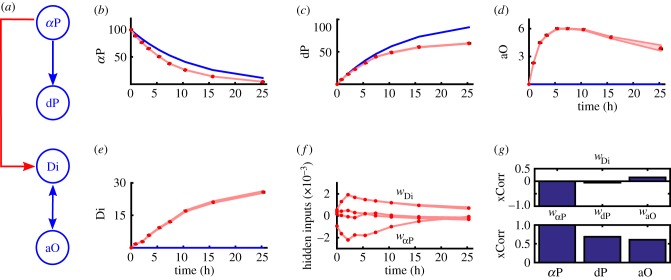
Reconstructing the hidden influence in a model of the dynamic *α*P isomerization [38]. (*a*) Reaction graph. The red arrow indicates a missing reaction. (*b*,*c*,*d*,*e*) Output measurements (black) compared with the posterior BDEN predictions (red) including 95% credible intervals and the nominal model (blue). (*f*) Estimates of the hidden influences (posterior mean) including 95% credible intervals. (*g*) Estimated relative cross-correlations (xCorr) of the hidden influence of *w*_*Di*_ to all estimated remaining hidden influences and state variables. BDEN is able to correctly detect the missing reaction.

To test BDEN the dimerization step was wrongly replaced with a simple reaction involving only aO. In consequence the interaction between *α*P and dP is completely independent from the interaction between aO and Di. The erroneous nominal system can be corrected by using BDEN as illustrated in figure 6. BDEN is able to correctly locate and add the falsely removed reaction.

### 3.4. Further examples with simulated data

In the following, we further illustrate the results obtained with BDEN for the G protein cycle in yeast, the UV-B signalling model and a generic gene-regulatory network using simulated data (2.5% noise level).

#### 3.4.1. G protein cycle in yeast

The heterotrimeric G protein cycle in yeast involves six species which are directly observable and coupled by several types of kinetics (for details see electronic supplementary material, §8) [34]. Experimental data at 8 time points were simulated by adding Gaussian distributed noise to the predicted values of the observable variables. We assumed a noise intensity of 2.5% relative to the mean of the related time series for each observable variable. The nominal system was generated by adding one additional ‘wrong’ interaction (between the ligand–receptor complex (LR) and the G protein_*α*_-inactive (GP_*αi*_)). Electronic supplementary material, figure S1, illustrates the ability of BDEN to localize and recover the wrong interaction within the nominal system.

#### 3.4.2. UV-B Signalling

As a more complex example, we simulated seven data points of the photomorphogenic UV-B signalling in plants [35]. The model of the photomorphogenic UV-B signalling in the model plant *Arabidopsis thaliana* consists of 11 species coupled by several different kinetic rate expressions and five observable variables as a combination of seven different species (for details see electronic supplementary material, §8). As the nominal system, we used the literature given model and included a missing link by removing one interaction which influences two different species. Observed data were simulated by adding Gaussian distributed noise to the predicted values of the observable variables. We assumed a noise intensity of 2.5% with respect to the mean of the related time series for each observable variable. As shown in electronic supplementary material, figure S2, the BDEN is able to detect the missing molecular interaction and correctly identifies the corresponding proteins.

#### 3.4.3. DREAM6 Challenge Network

To investigate the applicability of BDEN to gene-regulatory networks we took a model from the DREAM6 challenge [39]. The model consists of six genes and six proteins coupled by mass action and hill kinetics. In this model, all proteins and one mRNA species are assumed to be directly observable (for details see electronic supplementary material, §8) [39]. As the nominal system, we used the provided model and included one inhibitory mechanism. Observed data were simulated at five time points by adding Gaussian distributed noise to the predicted values of observable variables according to the original challenge [39]. BDEN is able to detect and correct the spurious interaction, as illustrated in electronic supplementary material, figure S3.

## 4. Conclusion

Mathematical modellers in systems biology are frequently confronted with incomplete knowledge and limited understanding of a complex biochemical system [9–11]. Consequently, there is a non-negligible chance that relevant molecular species are missed or interactions are misspecified [8,9]. Our proposed method addresses this issue by adopting a Bayesian framework which allows for inferring hidden influence variables, as well as estimating missing and wrong molecular interactions. It has been successfully validated in several real models as well as common network motifs. This was done with simulated as well as experimental data. Owing to the fully Bayesian formulation all model parameters are sampled. Furthermore, the Bayesian approach allows to assign confidence levels to predictions. Besides these general features of a fully probabilistic framework our newly proposed BDEN method seems to be more stable and more robust because within the Bayesian framework, we average over a large number of parameters and do not rely on stiff integration methods.

A unique feature of our new approach is the distinction between exogenous and endogenous hidden influences in the biological system, allowing for the detection of missing and misspecified equations in the ODE system. Altogether we thus see our BDEN method as a further step towards a better automated and more objective revision of ODE-based models in systems biology and possibly other fields, such as pharmacokinetics, earth science, robotics and engineering [12,20]. In that context, we emphasize again that BDEN is *not* designed to learn ODE systems purely from data and should thus not be confused with network reverse engineering methods [44]. Much more, the utility of BDEN is to ease identification of sources of errors in mechanism-based mathematical models.

## Supplementary Material

Supplementary Text

## Data accessibility

The source code (Matlab) with several examples is available online at http://www.abi.bit.uni-bonn.de/index.php?id=17.
