# Supplementary material for: A Bayesian approach to estimating hidden variables as well as missing and wrong molecular interactions in ordinary differential equation-based mathematical models
Source: J R Soc Interface. 2017 Jun 14;14(131):20170332. doi: 10.1098/rsif.2017.0332 (PMC5493809; doi:10.1098/rsif.2017.0332)
Supplement: Supplementary Text [file rsif20170332supp1.pdf]

Supplementary Text: A Bayesian approach to estimating hidden variables as well as missing and wrong molecular interactions in ordinary differential equation-based mathematical models

Benjamin Engelhardt[1,2,*], Maik Kschischo[3] and Holger Fröhlich[1,4]

[1]Rheinische Friedrich-Wilhelms-Universität Bonn, Algorithmic Bioinformatics, 53113 Bonn, Germany
[2]DFG Research Training Group 1873, Rheinische Friedrich-Wilhelms-Universität Bonn, Germany
[3]University of Applied Sciences Koblenz, RheinAhrCampus, Department of Mathematics and Technology, 53424 Remagen, Germany
[4]UCB Biosciences GmbH, 40789 Monheim, Germany
[*]To whom correspondence should be addressed.

June 6, 2017

1 Further results

List of Tables

S1 Performance of BDEN and DEN in dependence on the error of kinetic parameter estimates. . . . 2
S2 Performance of the BDEN in dependence on an increasing number of hidden influences for the G protein cycle in yeast relative to the number of nodes in the nominal model. . . . 3
S3 Performance of BDEN to correctly detect and classifyinteractions in dependence on the level of relative measurement noise. . . . 3
S4 Performance of BDEN to detect wrong and missing interactions in dependence on the error of the kinetic parameter estimate. . . . 4
S5 Performance of BDEN to correctly detect and classify interactions in dependence on the error of the kinetic parameter estimates. . . . 5

List of Figures

S1 Reconstructing the model error for the heterotrimeric G protein cycle in yeast. . . 5
S2 Reconstructing the model error for the photomorphogenic UV-B signaling in plants. 6
S3 Reconstructing the hidden influences in the gene-regulatory network. . . . 7

Model	Error Level	Method	AUC	BS
JAK-STAT	0%	BDEN	0.90 (0.15)	0.11 (0.11)
		DEN	0.60 (0.40)	0.16 (0.06)
	2.5%	BDEN	0.67 (0.15)	0.16 (0.10)
		DEN	0.51 (0.32)	0.32 (0.12)
	7.5%	BDEN	0.61 (0.18)	0.19(0.15)
		DEN	0.50 (0.33)	0.33 (0.13)
	12.5%	BDEN	0.59 (0.08)	0.26(0.16)
		DEN	0.43 (0.33)	0.33 (0.13)
G protein	0%	BDEN	1.00 (0.00)	0.04 (0.03)
		DEN	1.00 (0.00)	0.09 (0.02)
	2.5%	BDEN	1.00 (0.00)	0.05 (0.03)
		DEN	1.00 (0.00)	0.01 (0.01)
	7.5%	BDEN	0.96 (0.06)	0.12 (0.06)
		DEN	0.76 (0.20)	0.42 (0.05)
	12.5%	BDEN	0.95 (0.07)	0.15 (0.09)
		DEN	0.63 (0.23)	0.46 (0.05)
UV-B	0%	BDEN	0.91 (0.11)	0.19 (0.06)
		DEN	0.80 (0.19)	0.22 (0.08)
	2.5%	BDEN	0.78 (0.11)	0.14 (0.06)
		DEN	0.75 (0.14)	0.20 (0.06)
	7.5%	BDEN	0.76 (0.10)	0.15 (0.04)
		DEN	0.72 (0.15)	0.20 (0.06)
	12.5%	BDEN	0.73 (0.11)	0.16 (0.05)
		DEN	0.70 (0.19)	0.22 (0.07)
motifs	0%	BDEN	1.00 (0.00)	0.01 (0.00)
		DEN	0.90 (0.14)	0.11 (0.09)
	2.5%	BDEN	1.00 (< 0.01)	0.01 (0.01)
		DEN	0.93 (0.11)	0.06 (0.08)
	7.5%	BDEN	1.00 (< 0.01)	(< 0.01) (< 0.01)
		DEN	0.93 (0.11)	0.06 (0.08)
	12.5%	BDEN	0.99 (0.03)	0.01 (0.02)
		DEN	0.94 (0.10)	0.06 (0.08)

Table S1: Performance of BDEN and DEN in dependence on the error of the kinetic parameter estimates (median) for a fixed measurement noise of 2.5%. The median absolute deviations of the AUC (ROC) and Brier scores are given in brackets.

Percentage Hidden influences	AUC	BS
17%	1.00 (< 0.01)	0.02 (0.02)
33%	0.96 (0.06)	0.11 (0.09)
50%	0.86 (0.12)	0.22 (0.17)

Table S2: Performance of the BDEN in dependence on an increasing number of hidden influences for the G protein cycle in yeast relative to the number of nodes in the nominal model (median) for a fixed measurement noise of 2.5%. The median absolute deviations of the AUC (ROC) and Brier scores are given in brackets.

	Model	Noise	Class.	Overall
missing interaction	JAK-STAT	2.5%	1.00 (0.01)	0.80 (0.24)
		7.5%	1.00 (0.03)	0.70 (0.30)
		12.5%	0.91 (0.10)	0.70 (0.31)
	G protein	2.5%	1.00 (0.05)	0.71 (0.27)
		7.5%	0.93 (0.09)	0.58 (0.25)
		12.5%	0.85 (0.16)	0.54 (0.33)
	UV-B	2.5%	0.93 (0.09)	0.53 (0.15)
		7.5%	0.87 (0.15)	0.53 (0.09)
		12.5%	0.80 (0.23)	0.50 (0.14)

	Model	Noise	Class.	Overall
wrong interaction	JAK-STAT	2.5%	1.00 (0.02)	0.87 (0.19)
		7.5%	0.91 (0.06)	0.85 (0.19)
		12.5%	0.89 (0.12)	0.85 (0.29)
	G protein	2.5%	1.00 (0.01)	0.67 (0.19)
		7.5%	0.92 (0.14)	0.64 (0.10)
		12.5%	0.83 (0.25)	0.64 (0.18)
	UV-B	2.5%	0.91 (0.08)	0.68 (0.20)
		7.5%	0.87 (0.07)	0.61 (0.28)
		12.5%	0.83 (0.13)	0.50 (0.25)

Table S3: Performance of BDEN to correctly detect and classify interactions in dependence on the level of relative measurement noise (median AUC and MAD). The column "Class." reflects the accuracy for calling a correctly detected hidden influence as "wrong/missing stimulation" and "wrong/missing inhibition", respectively. In contrast, the column "Overall" reflects the accuracy for correctly detecting a hidden influence AND correctly classifying it as wrong/missing interaction AND calling it correctly "simulation" and "inhibition", respectively.

	Model	Error Level	AUC
missing interaction	JAK-STAT	0%	1.00 (0.00)
		2.5%	1.00 (0.00)
		7.5%	0.83 (0.28)
		12.5%	0.67 (0.44)
	G protein	0%	0.81 (0.19)
		2.5%	0.85 (0.21)
		7.5%	0.72 (0.40)
		12.5%	0.68 (0.43)
	UV-B	0%	1.00 (0.00)
		2.5%	1.00 (0.00)
		7.5%	0.75 (0.38)
		12.5%	0.65 (0.42)

	Model	Error Level	AUC
wrong interaction	JAK-STAT	0%	1.00 (0.00)
		2.5%	0.93 (0.13)
		7.5%	0.92 (0.14)
		12.5%	0.83 (0.28)
	G protein	0%	1.00 (0.00)
		2.5%	0.94 (0.10)
		7.5%	0.89 (0.20)
		12.5%	0.76 (0.34)
	UV-B	0%	0.81 (0.20)
		2.5%	0.84 (0.22)
		7.5%	0.83 (0.18)
		12.5%	0.74 (0.32)

Table S4: Performance of BDEN to detect wrong and missing interactions in dependence on the error of kinetic parameter estimates (median and MAD) for fixed measurement noise of 2.5%.

	Model	Error Level	Class.	Overall
Missing Interaction	JAK-STAT	0%	1.00 (0.01)	0.80 (0.24)
		2.5%	1.00 (0.08)	0.63 (0.19)
		7.5%	0.94 (0.10)	0.58 (0.23)
		12.5%	0.88 (0.12)	0.57 (0.26)
	G protein	0%	1.00 (0.05)	0.71 (0.27)
		2.5%	1.00 (0.03)	0.68 (0.32)
		7.5%	0.95 (0.11)	0.67 (0.27)
		12.5%	0.84 (0.13)	0.68 (0.25)
	UV-B	0%	0.93 (0.08)	0.53 (0.15)
		2.5%	0.93 (0.08)	0.52 (0.08)
		7.5%	0.85 (0.11)	0.52 (0.11)
		12.5%	0.78 (0.09)	0.52 (0.12)
Wrong Interaction	JAK-STAT	0%	1.00 (0.02)	0.87 (0.19)
		2.5%	0.99 (0.05)	0.90 (0.17)
		7.5%	0.99 (0.06)	0.82 (0.27)
		12.5%	0.91 (0.11)	0.77 (0.31)
	G protein	0%	1.00 (0.01)	0.67 (0.19)
		2.5%	1.00 (0.05)	0.62 (0.27)
		7.5%	0.93 (0.10)	0.52 (0.34)
		12.5%	0.81 (0.12)	0.48 (0.32)
	UV-B	0%	0.91 (0.08)	0.68 (0.20)
		2.5%	0.89 (0.09)	0.64 (0.18)
		7.5%	0.83 (0.12)	0.61 (0.22)
		12.5%	0.76 (0.16)	0.53 (0.32)

Table S5: Performance of BDEN to correctly detect and classify interactions in dependence on the error of the kinetic parameter estimates (median AUC and MAD) for a fixed measurement noise of 2.5%. The column "Class." reflects the accuracy for calling a correctly detected hidden influence as "wrong/missing stimulation" and "wrong/missing inhibition", respectively. In contrast, the column "Overall" reflects the accuracy for correctly detecting a hidden influence AND correctly classifying it as wrong/missing interaction AND calling it correctly "simulation" and "inhibition", respectively.

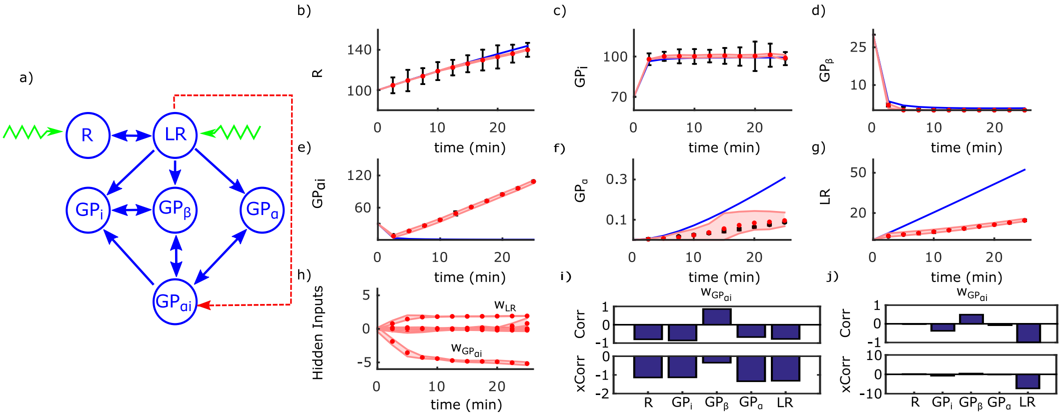

Figure S1: Reconstructing the hidden influence of the heterotrimeric G protein cycle in yeast Yi *et al.* (2003). (a) The reaction graph. (b,c,d,e,f,g) Synthetic measurements (black) compared to the posterior mean of BDEN predictions (red) including 95% credible intervals and the nominal model (blue). (h) Estimates of the hidden influences (posterior mean) including 95% credible intervals. (i) Estimated correlations (Corr) and cross-correlations (xCorr) of the hidden influence related to GP_α-inactive with all estimated state variables. (j) Estimated correlations and cross-correlations of the $w_{GP_{\alpha i}}$ related to GP_α-inactive with all remaining hidden influences.

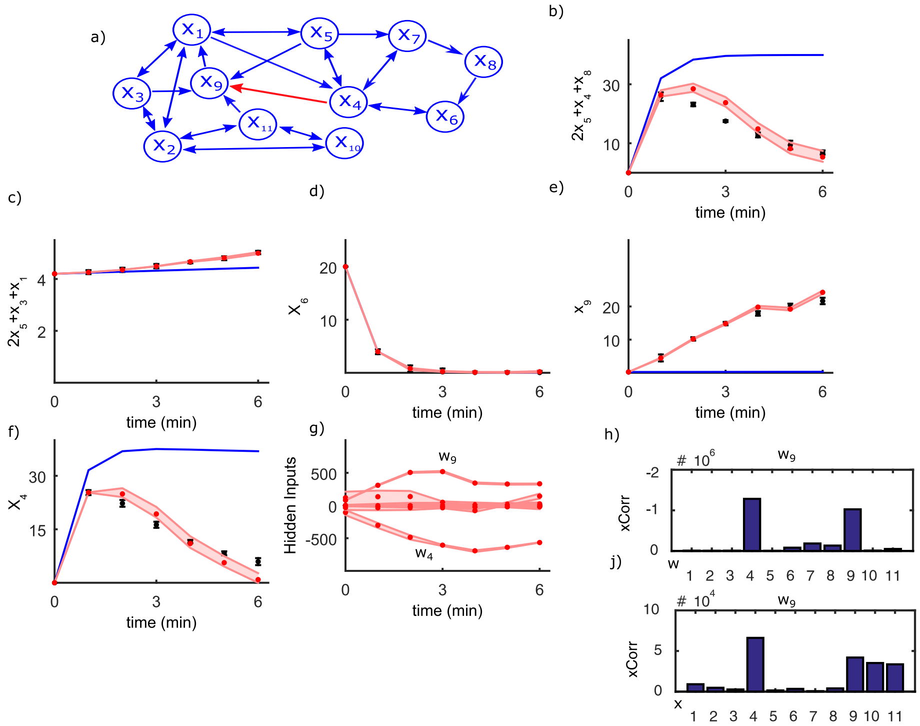

Figure S2: Reconstructing the model error for the photomorphogenic UV-B signaling (Ouyang *et al.*, 2014) in plants. (a) The reaction graph. (b,c,d,e) Synthetic measurements (black) compared to BDEN predictions (posterior mean) including 95% credible intervals and the nominal model (blue). (f) Posterior means of the hidden influences including 95% credible intervals. (g) Posterior means of the model variables including 95% credible intervals. (h) Estimated cross-correlations of all involved hidden influences with respect to w_9. (i) Estimated cross-correlations of all state variables with respect to w_9.

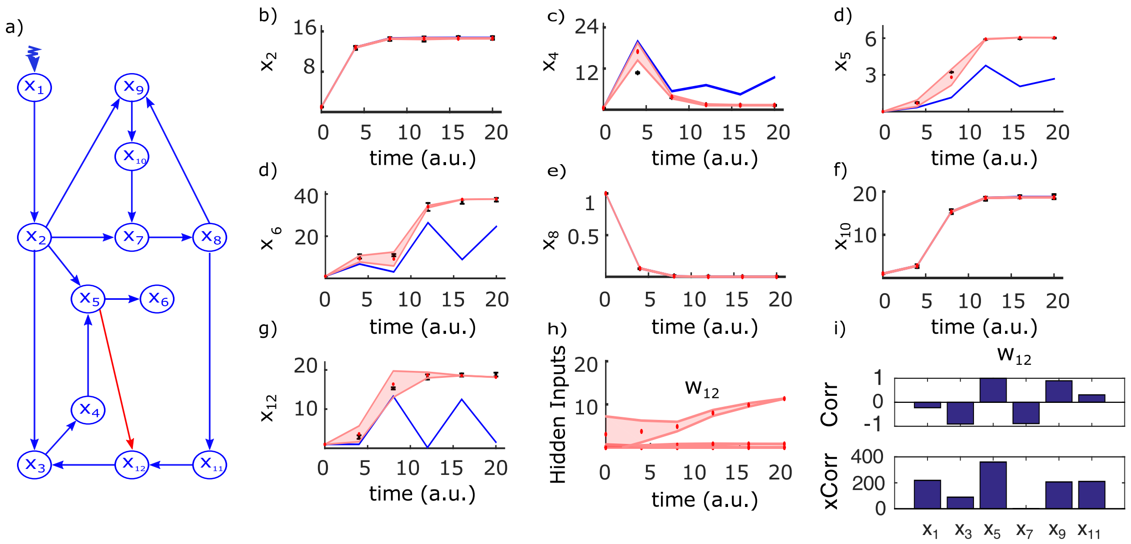

Figure S3: Reconstructing the hidden influence of the gene-regulatory network obtained from the DREAM6 challenge Meyer *et al.* (2014). (a) The interaction graph of the gene regulatory network including six proteins (even numbers) and related mRNAs (odd numbers). (b,c) Synthetic measurements of the protein concentrations (black) compared to the posterior mean of BDEN predictions (red) including 95% credible intervals and the nominal model (blue). (d) Synthetic measurements of the mRNA level (black) compared to the posterior mean of BDEN predictions (red) including 95% credible intervals and the nominal model (blue). (e,f,g) Synthetic measurements of the protein concentrations (black) compared to the posterior mean of BDEN predictions (red) including 95% credible intervals and the nominal model (blue). (h) Estimates of the hidden influences (posterior mean) including 95% credible intervals. (i) Estimated correlations (Corr) and cross-correlations (xCorr) of the hidden influence related to protein$_6$ (w_{12}) with all mRNAs ($x_1, x_3, x_5, x_7, x_9, x_{11}$) because in the gene-regulatory network direct protein-protein interactions are not considered Meyer *et al.* (2014). The highest correlation was found between protein$_6$ (x_{12}) and mRNA$_3$ (x_5). Hence, the spurious inhibition of protein$_6$ by mRNA$_3$ was correctly detected.

2 Details on simulation studies

Simulation of synthetic data was done as follows: First, a published ODE-based state-observation model with respective initial conditions was employed to generate noise-free observations at 6 time points, depending on the respective model (JAK-STAT - Section 8.4, UV-B signaling - Section 8.3, G protein cycling - Section 8.2, network motifs - Section 8.1). Subsequently Gaussian noise with fixed variance was added. Notably an (unrealistic) noise variance of zero is resulting into numerical problems for the BDEN and was thus not considered.

Hidden influences were simulated by removing one (Tables 2, 3, S1, S3, S4, S5) or up to three randomly picked (Table S2) state variables on the right hand side of the ODE system, yielding a wrong *nominal* ODE system. Likewise, wrong interactions were simulated by randomly adding one interaction on the right hand side of the ODE system. Missing interactions were simulated by randomly removing one of the existing interactions.

Due to the randomness of the process described above we repeated all simulations a number of times (Table S6). The exact number of repeats varies slightly to take into account the different number of combination possibilities. ODE systems with more state variables or allow for a larger number of possible combinations of hidden influence signals.

Table	Repeats
2	450
3	400
S1	450
S2	400
S3	350
S4	400
S5	350

Table S6: Number of independent simulation repeats for each of the presented results.

3 Additive hidden inputs and model error

The assumption of an additive hidden input incorporates many different types of model errors including parameter errors, hidden or misspecified interactions as well as inputs from exosystems. This can be seen as follows. Assume that the true system generating the data has the form

$$\begin{pmatrix} \dot{\boldsymbol{x}}(\boldsymbol{t}) \\ \dot{\boldsymbol{z}}(\boldsymbol{t}) \end{pmatrix} = \begin{pmatrix} \boldsymbol{\phi}(\boldsymbol{x}(t), \boldsymbol{z}(t), \boldsymbol{u}(t)) \\ \boldsymbol{\psi}(\boldsymbol{x}(t), \boldsymbol{z}(t), \boldsymbol{u}(t)) \end{pmatrix} \quad (1a)$$

$$\boldsymbol{y}(t) = \boldsymbol{h}(\boldsymbol{x}(t)) . \quad (1b)$$

Here, $\boldsymbol{x}(t) = (x_1(t), \dots, x_N(t))' \in \mathbb{R}^N$ is the state vector also included in the nominal model

$$\dot{\boldsymbol{x}}(t) = \boldsymbol{f}(\boldsymbol{x}(t), \boldsymbol{u}(t)) \quad (2a)$$

$$\boldsymbol{y}(t) = \boldsymbol{h}(\boldsymbol{x}(t)) , \quad (2b)$$

which represents our current knowledge or assumptions about the true system. We assume that the output map $\boldsymbol{h}$ is exactly known and depends only on $\boldsymbol{x}$, so (2b) and (1b) have the same form. The exostate $\boldsymbol{z}(t) = (z_1(t), \dots, z_M(t))'$ in the true system (1) represents dynamic variables ignored in the nominal model. Let us denote the solution of the true system (1a) as

$$\boldsymbol{\nu}(t) = \begin{pmatrix} \boldsymbol{x}(t) \\ \boldsymbol{z}(t) \end{pmatrix} \quad \text{with initial condition} \quad \boldsymbol{\nu}(t_0) = \begin{pmatrix} \boldsymbol{x}(t_0) \\ \boldsymbol{z}(t_0) \end{pmatrix} . \quad (3)$$

Now we compare the functions $\boldsymbol{\phi}$ and $\boldsymbol{f}$ along the true state trajectory (3) and define

$$\boldsymbol{w}(t) := \dot{\boldsymbol{x}}(t) - \boldsymbol{f}\left(\boldsymbol{x}(t), \boldsymbol{u}(t)\right) = \boldsymbol{\phi}\left(\boldsymbol{x}(t), \boldsymbol{z}(t), \boldsymbol{u}(t)\right) - \boldsymbol{f}\left(\boldsymbol{x}(t), \boldsymbol{u}(t)\right).$$

Thus,

$$\dot{\boldsymbol{x}}(t) = \boldsymbol{f}\left(\boldsymbol{x}(t), \boldsymbol{u}(t)\right) + \boldsymbol{w}(t). \tag{4}$$

represents the true dynamics $\boldsymbol{x}(t)$ through the nominal system $\boldsymbol{f}$ and the hidden input $\boldsymbol{w}(t)$. Note that we have suppressed the dependence on the parameters in the notation. However, this can be seen as part of the systems specification, i.e., as defining the properties of $\boldsymbol{f}$, $\boldsymbol{\phi}$ and $\boldsymbol{\psi}$, respectively. Thus, model errors can be represented by hidden (unknown) inputs.

4 Full derivation of Equation (6)

According to Zacher *et al.* (2012), Eq. (6) can be derived as follows:

Given $y_{k,l}\left|\boldsymbol{x}_l, \xi^2_{k,l}\right. \sim N\left(y_{k,l}\left|\boldsymbol{x}_l, 1/\tau_{k,l}\right.\right)$ with unknown $\tau = \frac{1}{\xi^2_{k,l}}$ and $\tau \sim G\left(\alpha, \beta\right)$, we obtain

$$\begin{aligned}
p\left(\boldsymbol{y}_{k,l}\left|\boldsymbol{x}_l, \alpha, \beta\right.\right) &\propto \int p\left(y_{k,l}\left|\boldsymbol{x}_l, \xi^2_{k,l}\right.\right) \times p\left(\left.\xi^2_{k,l}\right| \alpha, \beta\right) d\xi^2_{k,l} \\
&= \int N\left(y_{k,l}\left|\boldsymbol{x}_l, \tau_{k,l}\right.\right) \times G\left(\left.\tau_{k,l}\right| \alpha, \beta\right) d\tau_{k,l} \\
&= \int \frac{\beta^\alpha}{\Gamma\left(\alpha\right)} \tau^{\alpha-1} \exp\left(-\tau\beta\right) \left(\frac{\tau}{2\pi}\right)^{\frac{1}{2}} exp\left(-\frac{\tau}{2}\left(y_{k,l} - h_k\left(\boldsymbol{x}_l\right)\right)^2\right) d\tau_{k,l} \\
&= \frac{\beta^\alpha}{\Gamma\left(\alpha\right)} \frac{1}{\sqrt{2\pi}} \int \tau^{\alpha+\frac{1}{2}-1} \exp\left(-\frac{\tau\left(2\beta + \left(y_{k,l} - h_k\left(\boldsymbol{x}_l\right)\right)^2\right)}{2}\right) d\tau_{k,l} \\
&= \frac{\beta^\alpha}{\Gamma\left(\alpha\right)} \frac{1}{\sqrt{2\pi}} \frac{\Gamma\left(\alpha + \frac{1}{2}\right)}{\left(\beta + \frac{1}{2}\left(y_{k,l} - h_k\left(\boldsymbol{x}_l\right)\right)^2\right)^{\alpha+\frac{1}{2}}} \\
&= \frac{\Gamma\left(\alpha + \frac{1}{2}\right)}{\Gamma\left(\alpha\right)} \frac{1}{\left(2\pi\beta\right)^{\frac{1}{2}}} \frac{1}{\left(1 + \frac{1}{2\beta}\left(y_{k,l} - h_k\left(\boldsymbol{x}_l\right)\right)^2\right)^{\alpha+\frac{1}{2}}}
\end{aligned}$$

5 Methodology

The full hierarchical model of the Bayesian elastic-net is given by (Zou and Hastie, 2005; Kyung *et al.*, 2010):

$$
\begin{aligned}
\omega_{i,l} \left| \sigma^2, \boldsymbol{\tau}^2, \lambda_2, \omega_{i,l-1} \right. &\sim N\left(\omega_{i,l-1}, \frac{\sigma^2 \tau_i^2}{\lambda_2 \tau_i^2 + 1}\right) \\
\boldsymbol{\tau}^2 \left| \boldsymbol{\lambda}_1^2 \right. &\sim \prod_{i=1}^{N} \left[\frac{\lambda_{1,i}^2}{2} \exp\left(-\frac{\lambda_{1,i}^2 \tau_i^2}{2} \right) \right], \quad \tau_1^2, ..., \tau_N^2 > 0 \\
\boldsymbol{\lambda}_1^2 &\sim \prod_{i=1}^{N} \left[\frac{\delta_{1,i}^{r_{1,i}}}{\Gamma(r_{1,i})} \left(\lambda_{1,i}^2\right)^{r_{1,i}-1} \exp\left(-\delta_{1,i}\lambda_{1,i}^2\right) \right] \\
\lambda_2 &\sim \frac{\delta_2^{r_2}}{\Gamma(r_2)} \lambda_2^{r_2-1} \exp\left(-\delta_2 \lambda_2\right) \\
\sigma^2 &\sim p\left(\sigma^2\right), \quad \sigma^2 > 0.
\end{aligned}
$$

For σ^2 we chose a standard non-informative, improper and scale-invariant prior $p\left(\sigma^2\right) \propto \sigma^{-2}$ (Park and Casella, 2008; Kyung *et al.*, 2010). In contrast to the variance of the measurement noise $\boldsymbol{\xi}_l$, σ^2 represents the variance of the hidden influences. The parameters $\boldsymbol{\lambda}_1$ and λ_2 control the sparsity and smoothness of the resulting hidden influence dynamics, respectively. Please note that the parameter $\boldsymbol{\lambda}_1$ in contrast to λ_2 is controlled by an additional hyper-parameter $\boldsymbol{\tau}^2$. For $l = 1$ the full Bayesian elastic-net prior corresponds to a product of a Gaussian and Laplace distribution (Zou and Hastie, 2005; Kyung *et al.*, 2010).

6 Sampling Algorithm

Algorithm 1: Pseudo Code

for $l \leftarrow 1$ **to** T **do**

  for $s \leftarrow 1$ **to** S **do**

1. $i \leftarrow$ random species
   $w^s_{i,t_l} \leftarrow w_{i,l-1} + N(0, J)$
   Accept $w^s_{i,l}$ with probability $\Phi\left(\boldsymbol{w}^s_l \middle| \boldsymbol{w}^{(s-1)}_l\right)$, otherwise set $w^s_{i,l} \leftarrow w^{(s-1)}_{i,l}$
2. Draw $\sigma^2 \sim inverseGamma\left(\frac{n}{2}, \frac{\zeta + \sum_{i=1}^{N}(w_{i,l}-w_{i,l-1})^2 \frac{\lambda_2\tau_i^2+1}{\tau_i^2}}{2}\right)$
3. Draw $\tau_i^{-2} \sim inverseGaussian\left(\sqrt{\frac{\lambda^2_{1,i}\sigma^2}{\omega_{i,l}-\omega_{i,l-1}}}, \lambda^2\right)$ for $i = 1, ..., N$
4. Draw $\lambda^2_{1,i} \sim Gamma\left(r_{1,i}+1, \frac{\tau_i^2}{2} + \delta_{1,i}\right)$ for $i = 1, ..., N$
5. Draw $\lambda_2 \sim Gamma\left(r_2 + \frac{n}{2}, \frac{1}{2\sigma^2}\|\boldsymbol{\omega}_l - \boldsymbol{\omega}_{l-1}\|_2^2 + \delta_2\right)$

  $\boldsymbol{w}_l = \frac{1}{S}\sum_{s=1}^{S}\omega^s_l;$

  $x_l = \int_{t_{l-1}}^{t_l} \boldsymbol{f}\left(\boldsymbol{x}(t'_l)\,\boldsymbol{u}(t'_l)\right) + \boldsymbol{w}(t'_l)\,dt'\,\Big|_{x_{l-1}}, \quad \boldsymbol{x}_0 = \eta$

$$\Phi\left(\boldsymbol{w^s}_l \middle| \boldsymbol{w}^{(s-1)}_l\right) = \min\left\{1, \frac{p(\boldsymbol{y}_l|\boldsymbol{x}_l, \alpha, \beta)}{p(\boldsymbol{y}_l|\boldsymbol{x}_l, \alpha, \beta)} \times \frac{p(\boldsymbol{x}_l|\boldsymbol{x}_{l-1}, \boldsymbol{w}^s_l, \boldsymbol{w}_{l-1})}{p(\boldsymbol{x}_l|\boldsymbol{x}_{l-1}, \boldsymbol{w}^{s-1}_l, \boldsymbol{w}_{l-1})} \times \frac{p(\boldsymbol{w}^s_l|\theta)}{p(\boldsymbol{w}^{s-1}_l|\theta)}\right\}$$
$$= \min\left\{1, \prod_{i=1}^{K}\left(\frac{2\beta + (y_{k,l} - h_k(\boldsymbol{x}_l))^2}{2\beta + (y_{k,l} - h_k(\boldsymbol{x}_l))^2}\right)^{\alpha+\frac{1}{2}} \times \frac{p(\boldsymbol{w}^s_l|\sigma^2, \boldsymbol{\tau}^2, \lambda_2, \omega_{i,l-1})}{p(\boldsymbol{w}^{s-1}_l|\sigma^2, \boldsymbol{\tau}^2, \lambda_2, \omega_{i,l-1})}\right\}$$

Step 1 is given by a Metropolis-Hastings move (Algorithm 2) with respect to J (Brooks, 1998). Avoiding autocorrelation, the mean for $\boldsymbol{w}_l$ is adapted via thinning (Gelman *et al.*, 2013).

Algorithm 2: Independent MH

for $s' \leftarrow 1$ **to** S' **do**

  Draw $I \sim U\{1, N\}$

  Draw $\omega^*_{i=I,l} \sim N(\omega_{i=I,l-1}, J)$

  $\omega^*_{i \neq I,l} = \omega^{(s'-1)}_{i \neq I,l}$

  if $\Phi\left(\boldsymbol{w}^{s'}{}_l \middle| \boldsymbol{w}^{(s'-1)}_l\right) \geq \alpha$ **then** $\omega^{s'}_{i,l} = \omega^*_{i,l}$

  else $\omega^{s'}_{i,l} = \omega^{(s'-1)}_{i,l}$

$\boldsymbol{w}_l = \frac{1}{S'} \sum_{s'=1}^{S'} \omega^{s'}_l$

7 Hyper-parameter Settings

Following an empirical Bayesian approach, α and β in the variance prior are estimated by fitting the inverse sample variance distribution via maximum likelihood. An initial value for σ^2 can be approximated by the maximal distance between two subsequent $\boldsymbol{w}(t_l)$ in the time series

$$\max_{k,l>1}\left(|\omega_i(t_l)| - |\omega_i(t_{l-1})|\right) \propto \max_{k,l>1}\left(\left|\frac{d\left(y_k(t_l) - h_k(\boldsymbol{x}(t_l))\right)}{dt_l}\right| - \left|\frac{d\left(y_k(t_{l-1}) - h_k(\boldsymbol{x}(t_{l-1}))\right)}{dt_{l-1}}\right|\right). \tag{5}$$

We advise to include a parameter ζ to omit values next to zero for σ^2, which would lead to numerical issues. Here a good conservative choice is also given by Equation (5).

The parameter J of the candidate distribution $\pi \sim N(0, J)$ can be defined as the maximum of the mean largest value of the approximated hidden influence with respect to the least square error. To increase performance we suggest a burn-in phase of about 33% of the total number of iterations, which was set to $1500 \times N$ as a compromise between computational time and sampling quality. The number of Metropolis-Hastings moves was set to be ten times larger than the number of Gibbs steps.

Due to the low number of measurement points and their variance we introduce the additional parameter $\gamma \leq 1$ which means that the measurement noise is reduced by γ and $\xi_{i,l}$ is re-parametrized as

$$\xi^2{}_{i,l} \sim IG\left(\alpha, \frac{\beta}{\gamma}\right). \tag{6}$$

Since the re-parametrized variance estimator yields a sharper prior distribution, it is more conservative.

The parameters $\boldsymbol{\lambda}_1$ and λ_2 themselves depend on hyper-parameters, which can be set in a non-informative manner or with respect to prior knowledge about the degree of shrinkage and smoothness of the hidden influences (Kyung *et al.*, 2010).

8 Model Details

8.1 Motifs

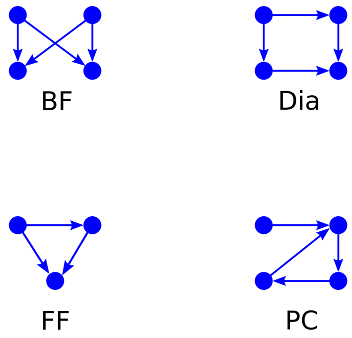

Graphical representation of the investigated Bi-Fan motif (BF), Diamond motif (Dia), Feed-Forward loop (FF) and the Protein cascade (PC) (Milo, 2002).

For all motifs (see Milo (2002)) we used the monotonic function

$$u(t) = \left(1 - \frac{1}{1+t}\right)$$

as known input stimulus and the initial condition $\boldsymbol{x}(0) = \mathbf{0}$ for all state variables with $t = [0, 1]$. The system is assumed to be fully observable.

8.1.1 Bi-fan

$$\begin{aligned}
\dot{x}_1(t) &= u(t) - x_1(t) \\
\dot{x}_2(t) &= u(t) - x_2(t) \\
\dot{x}_3(t) &= x_2(t) + x_1(t) - x_3(t) \\
\dot{x}_4(t) &= x_2(t) + x_1(t) - x_4(t)
\end{aligned}$$

8.1.2 Diamond

$$\begin{aligned}
\dot{x}_1(t) &= u(t) - x_1(t) \\
\dot{x}_2(t) &= x_1(t) - x_2(t) \\
\dot{x}_3(t) &= x_1(t) - x_3(t) \\
\dot{x}_4(t) &= x_2(t) + x_3(t) - x_4(t)
\end{aligned}$$

8.1.3 Feed-forward Loop

$$\begin{aligned}
\dot{x}_1(t) &= u(t) - x_1(t) \\
\dot{x}_2(t) &= x_1(t) - x_2(t) \\
\dot{x}_3(t) &= x_2(t) + x_1(t) - x_3(t)
\end{aligned}$$

8.1.4 Protein Cascade

$$\begin{aligned}
\dot{x}_1(t) &= u(t) - x_1(t) \\
\dot{x}_2(t) &= \frac{x_1(t)}{1 + x_4} - x_2(t) \\
\dot{x}_3(t) &= x_2(t) - x_3(t) \\
\dot{x}_4(t) &= x_3(t) - x_4(t)
\end{aligned}$$

8.2 G protein signaling model

The model for the heterotrimeric G protein cycle in yeast (Yi *et al.*, 2003) was downloaded from the BioModels Database (Li *et al.*, 2010) (BIOMD0000000072). The system is assumed to be fully observable.

$$
\begin{aligned}
\frac{\mathrm{d}([R])}{\mathrm{d}t} &= -\left(3.32e^{-18}\right)[L]_{const.}\,[\mathrm{R}] + 0.01\,[RL] - 4 - 0.0004\,[R] \\
\frac{\mathrm{d}([\mathrm{GP_i}])}{\mathrm{d}t} &= [\mathrm{GP}_{\alpha\mathrm{i}}]\,[GP_\beta] - \left(1e^{-05}\right)[RL]\,[\mathrm{GP_i}] \\
\frac{\mathrm{d}([GP_\beta])}{\mathrm{d}t} &= -[\mathrm{GP}_{\alpha\mathrm{i}}]\,[GP_\beta] + \left(1e^{-05}\right)[RL]\,[\mathrm{GP_i}] \\
\frac{\mathrm{d}\left([\mathrm{GP}_{\alpha_\mathrm{i}}]\right)}{\mathrm{d}t} &= -[\mathrm{GP}_{\alpha\mathrm{i}}]\,[GP_\beta] + 0.11\,[\mathrm{GP}_\alpha] \\
\frac{\mathrm{d}([\mathrm{GP}_\alpha])}{\mathrm{d}t} &= \left(1e^{-05}\right)[RL]\,[\mathrm{GP_i}] - 0.11\,[\mathrm{GP}_\alpha] \\
\frac{\mathrm{d}([RL])}{\mathrm{d}t} &= \left(3.32e^{-18}\right)[L]_{const.}\,[R] - 0.01\,[RL] - 0.004\,[RL]
\end{aligned}
$$

The initial conditions are given by

$$
\begin{aligned}
[R]_0 &= 100\ item\backslash l; & [\mathrm{GP}_{\alpha\mathrm{i}}]_0 &= 30\ item\backslash l; \\
[\mathrm{GP_i}]_0 &= 70\ item\backslash l; & [\mathrm{GP}_\alpha]_0 &= 0\ item\backslash l; \\
[GP_\beta]_0 &= 30\ item\backslash l; & [RL]_0 &= 0\ item\backslash l; \\
[L]_{const.} &= 0.02\ item\backslash l,
\end{aligned}
$$

and $t = [0, 25]$.

8.3 UV-B Network model

The model equations for the UV-B signaling network (Ouyang *et al.*, 2014) were obtained from the BioModels Database (Li *et al.*, 2010), see BIOMD0000000545.

$$
\begin{aligned}
\frac{d\,[\mathrm{CS}]}{dt} =& -2 \cdot ka_1 \cdot [\mathrm{CS}]^2 \cdot [\mathrm{UVR8M}]^2 + 2kd_1 \cdot [\mathrm{UCS}] \\
&+ ks_1 \cdot (1 + UV \cdot n_3 \cdot ([\mathrm{HY5}] + FHY3)) \\
&- kdr_1 \cdot (1 + (n_1 \cdot UV)) \cdot [\mathrm{CS}] - kd_2 \cdot [\mathrm{CDCS}] \\
&- 2 \cdot ka_2 \cdot [\mathrm{CS}]^2 \cdot [\mathrm{CD}] \\
\frac{d\,[\mathrm{CD}]}{dt} =& - ka_2 \cdot [\mathrm{CS}]^2 \cdot [\mathrm{CD}] + kd_2 \cdot [\mathrm{CDCS}] \\
&+ ka_4 \cdot [\mathrm{CD}] \cdot [\mathrm{DWD}] + kd_4 \cdot [\mathrm{CDW}] \\
\frac{d\,[\mathrm{CDCS}]}{dt} =& - kd_2 \cdot [\mathrm{CDCS}] + ka_2 \cdot [\mathrm{CS}]^2 \cdot [\mathrm{CD}] \\
\frac{d\,[\mathrm{UVR8M}]}{dt} =& -2 \cdot k_1 \cdot [\mathrm{UVR8M}]^2 + 2 \cdot k_2 \cdot [\mathrm{UVR8D}] \\
&- 2 \cdot ka_1 \cdot [\mathrm{CS}]^2 \cdot [\mathrm{UVR8M}]^2 + 2 \cdot kd_1 \cdot [\mathrm{UCS}] \\
&- ka_3 \cdot [\mathrm{UVR8M}] \cdot [\mathrm{RUP}] \\
\frac{d\,[\mathrm{UCS}]}{dt} =& - kd_1 \cdot [\mathrm{UCS}] + ka_1 \cdot [\mathrm{CS}]^2 \cdot [\mathrm{UVR8M}]^2 \\
\frac{d\,[\mathrm{UVR8D}]}{dt} =& - k_2 \cdot [\mathrm{UVR8D}] + k_1 \cdot [\mathrm{UVR8M}]^2 + kd_3 \cdot [\mathrm{UR}]^2 \\
\frac{d\,[\mathrm{RUP}]}{dt} =& - ka_3 \cdot [\mathrm{UVR8M}] \cdot [\mathrm{RUP}] + ks_2 \cdot (1 + UV \cdot [\mathrm{UCS}]) \\
&- kdr2 \cdot [\mathrm{RUP}] + (2) \cdot kd_3 \cdot [\mathrm{UR}]^2 \\
\frac{d\,[\mathrm{UR}]}{dt} =& -2 \cdot kd_3 \cdot [\mathrm{UR}]^2 + ka_3 \cdot [\mathrm{UVR8M}] \cdot [\mathrm{RUP}] \\
\frac{d\,[\mathrm{HY5}]}{dt} =& - kdr3 \cdot \left(\frac{[\mathrm{CDCS}]}{kdr_{3a} + [\mathrm{CDCS}]} + \frac{[\mathrm{CDW}]}{kdr_{3b} + [\mathrm{CDW}]} \right) \cdot [\mathrm{HY5}] \\
&+ ks3_p \cdot (1 + n_2 \cdot UV) - kdr3 \cdot \left(\frac{[\mathrm{UCS}]}{ksr + [\mathrm{UCS}]} \right) \cdot [\mathrm{HY5}] \\
\frac{d\,[\mathrm{DWD}]}{dt} =& - ka_4 \cdot [\mathrm{CD}] \cdot [\mathrm{DWD}] + kd_4 \cdot [\mathrm{CDW}] \\
\frac{d\,[\mathrm{CDW}]}{dt} =& - kd_4 \cdot [\mathrm{CDW}] + ka_4 \cdot [\mathrm{CD}] \cdot [\mathrm{DWD}]
\end{aligned}
$$

$$
\begin{aligned}
UM_{Total} &= 2 \cdot [\mathrm{UCS}] + [\mathrm{UVR8M}] + [\mathrm{UR}] \\
COP1_{Total} &= 2 \cdot [\mathrm{UCS}] + 2 \cdot [\mathrm{CDCS}] + [\mathrm{CS}] \\
UVR8D_{obs.} &= [\mathrm{UVR8D}] \\
HY5_{obs.} &= [\mathrm{HY5}] \\
UVR8M_{obs} &= [\mathrm{UVR8M}]
\end{aligned}
$$

$$
\begin{aligned}
[\mathrm{CS}] &= x_1; & [\mathrm{UVR8M}] &= x_4; & [\mathrm{RUP}] &= x_7; & [\mathrm{DWD}] &= x_{10}; \\
[\mathrm{CD}] &= x_2; & [\mathrm{UCS}] &= x_5; & [\mathrm{UR}] &= x_8; & [\mathrm{CDW}] &= x_{11}; \\
[\mathrm{CDCS}] &= x_3; & [\mathrm{UVR8D}] &= x_6; & [\mathrm{HY5}] &= x_9 & &
\end{aligned}
$$

The initial conditions are given by

$$
\begin{aligned}
[\mathrm{CS}]_0 &= 0.2mol & [\mathrm{RUP}]_0 &= 0mol \\
[\mathrm{CD}]_0 &= 10mol & [\mathrm{UR}]_0 &= 0mol \\
[\mathrm{CDCS}]_0 &= 2mol & [\mathrm{HY5}]_0 &= 0.25mol \\
[\mathrm{UVR8M}]_0 &= 0mol & [\mathrm{DWD}]_0 &= 20mol \\
[\mathrm{UCS}]_0 &= 0mol & [\mathrm{CDW}]_0 &= 0mol \\
[\mathrm{UVR8D}]_0 &= 20mol, & &
\end{aligned}
$$

and $t = [0, 6]$.

8.4 JAK-STAT model

The measurements for the JAK-STAT system were obtained from http://webber.physik.uni-freiburg.de/~jeti/PNAS_Swameye_Data. Mass conservation was accounted for by the constraint $2x_4(t) + 2x_3(t) + x_1(t) + x_2(t) = \text{const.}$ for all t (Swameye *et al.*, 2003; Raue *et al.*, 2009). The nominal model is given by (Swameye *et al.*, 2003):

$$
\begin{aligned}
\dot{x}_1 &= -\psi_1 x_1 u \\
\dot{x}_2 &= \psi_1 x_1 u - 2\psi_2 x_2^2 \\
\dot{x}_3 &= \psi_2 x_2^2 - \psi_3 x_3 \\
\dot{x}_4 &= \psi_3 x_3 \\
y_2 &= \psi_5 \left(x_1 + x_2 + 2x_3 \right) \\
y_1 &= \psi_4 \left(x_2 + 2x_3 \right)
\end{aligned}
$$

$$
\begin{aligned}
u &= \text{Erythropoietin Receptor} \\
x_1 &= \mathrm{STAT5} \\
x_2 &= \mathrm{STAT5_p} \\
x_3 &= \mathrm{STAT5_{di}} \\
x_4 &= \mathrm{STAT5_n} \\
y_1 &= \text{total STAT5} \\
y_2 &= \text{total } \mathrm{STAT5_p},
\end{aligned}
$$

and $t = [0, 60]$.

8.5 Model of information processing at EpoR

The Model of the information processing at EpoR was obtained from Becker *et al.* (2010). Data was obtained from dMod.

$$\begin{aligned}
\dot{x}_1 =& k_t \cdot B_{max} - k_t \cdot x_1 - k_on \cdot x_1 \cdot x_2 + k_{off} \cdot x_3 + k_{ex} \cdot x_4 \\
\dot{x}_2 =& -k_{on} \cdot x_1 \cdot x_2 + k_{off} \cdot x_3 + k_{ex} \cdot x_4 \\
\dot{x}_3 =& k_{on} \cdot x_1 \cdot x_2 - k_{off} \cdot x_3 - k_e \cdot x_3 \\
\dot{x}_4 =& k_e \cdot x_3 - k_{ex} \cdot x_4 - k_{di} \cdot x_4 - k_{de} \cdot x_4 \\
\dot{x}_5 =& k_{di} \cdot x_4 \\
\dot{x}_6 =& k_{de} \cdot x_4 \\
y_2 =& \psi_1 (x_2 + 2x_6) \\
y_1 =& \psi_2 (x_3) \\
y_3 =& \psi_3 (x_4 + x_5)
\end{aligned}$$

$$\begin{aligned}
x_1 &= \text{EpoR} \\
x_2 &= \text{Epo} \\
x_3 &= \text{Epo-EpoR} \\
x_4 &= \text{Epo-EpoR}_\text{i} \\
x_5 &= \text{dEpo}_\text{i} \\
x_6 &= \text{dEpo}_\text{e} \\
y_1 &= \text{Epo concentration in medium} \\
y_2 &= \text{Epo concentration on surface} \\
y_3 &= \text{Epo concentration in cells,}
\end{aligned}$$

and $t = [0, 300]$.

8.6 Model of α-Pinene isomerization

The model of the thermal isomerization of α-Pinene in the liquid phase was obtained from Fuguitt and Hawkins (1947).

$$\begin{aligned}
\dot{x}_1 =& -(p_1 + p_2) \cdot x_1 \\
\dot{x}_2 =& p_1 \cdot x_1 \\
\dot{x}_3 =& p_2 \cdot x_1 - (p_3 + p_4) \cdot x_3 + p_5 \cdot x_4 \\
\dot{x}_4 =& p_4 \cdot x_3 - p_5 \cdot x_4
\end{aligned}$$

$$\begin{aligned}
x_1 &= \alpha\text{-Pinene} \\
x_2 &= \text{Dipentene} \\
x_3 &= \text{Dimer} \\
x_4 &= \text{allo-ocimene,}
\end{aligned}$$

and $t = [0, 25]$.

8.7 Gene-regulatory network

The gene-regulatory network was obtained from DREAM6 challenge Meyer *et al.* (2014). For further information about this challenge we refer the reader to the DREAM6 challenge.

$$\begin{aligned}
\dot{x}_1 =& pro1_{strength} - mrna1_{degradation-rate} \cdot x_1 \\
\dot{x}_2 =& rbs1_{strength} \cdot x_1 - p_{degradation-rate} \cdot x_2 \\
\dot{x}_3 =& pro2_{strength} \cdot \frac{\left(\frac{x_1}{v2_{Kd}}\right)^{v2_h}}{1+\left(\frac{x_2}{v2_{Kd}}\right)^{v2_h}} \cdot \frac{1}{1+\left(\frac{x_6}{v5_{Kd}}\right)^{v5_h}} - mrna2_{degradation-rate} \cdot x_3 \\
\dot{x}_4 =& rbs2_{strength} \cdot x_3 - p_{degradation-rate} \cdot x_4 \\
\dot{x}_5 =& pro3_{strength} \cdot \frac{\left(\frac{x_1}{v3_{Kd}}\right)^{v3_h}}{1+\left(\frac{x_2}{v3_{Kd}}\right)^{v3_h}} \cdot \frac{1}{1+\left(\frac{x_2}{v4_{Kd}}\right)^{v4_h}} - mrna3_{degradation-rate} \cdot x_5 \\
\dot{x}_6 =& rbs3_{strength} \cdot x_5 - p_{degradation-rate} \cdot x_6 \\
\dot{x}_7 =& pro4_{strength} \cdot \frac{\left(\frac{x_1}{v1_{Kd}}\right)^{v1_h}}{1+\left(\frac{x_2}{v1_{Kd}}\right)^{v1_h}} \cdot \frac{1}{1+\left(\frac{x_5}{v8_{Kd}}\right)^{v8_h}} - mrna4_{degradation-rate} \cdot x_7 \\
\dot{x}_8 =& rbs4_{strength} \cdot x_7 - p_{degradation-rate} \cdot x_8 \\
\dot{x}_9 =& pro5_{strength} \cdot \frac{1}{1+\left(\frac{x_4}{v6_{Kd}}\right)^{v6_h}} - mrna5_{degradation-rate} \cdot x_9 \\
\dot{x}_{10} =& rbs5_{strength} \cdot x_9 - p_{degradation-rate} \cdot x_{10} \\
\dot{x}_{11} =& pro6_{strength} \cdot \frac{1}{1+\left(\frac{x_4}{v7_{Kd}}\right)^{v7_h}} - mrna6_{degradation-rate} * x_{11} \\
\dot{x}_{12} =& rbs6_{strength} \cdot x_{11} - p_{degradation-rate} \cdot x_{12}
\end{aligned}$$

$$\begin{aligned}
y_1 &= \mathrm{pp1_{mrna}} \\
y_2 &= \mathrm{p1} \\
y_3 &= \mathrm{p2} \\
y_4 &= \mathrm{p3} \\
y_5 &= \mathrm{p4} \\
y_6 &= \mathrm{p5} \\
y_7 &= \mathrm{p6}
\end{aligned}$$

$$\begin{aligned}
x_1 &= \mathrm{pp1_{mrna}} \\
x_2 &= \mathrm{p1} \\
x_3 &= \mathrm{pp2_{mrna}} \\
x_4 &= \mathrm{p2} \\
x_5 &= \mathrm{pp3_{mrna}} \\
x_6 &= \mathrm{p3} \\
x_7 &= \mathrm{pp4_{mrna}} \\
x_8 &= \mathrm{p4} \\
x_9 &= \mathrm{pp5_{mrna}} \\
x_{10} &= \mathrm{p5} \\
x_{11} &= \mathrm{pp6_{mrna}} \\
x_{12} &= \mathrm{p6}
\end{aligned}$$

The initial conditions for the proteins $p1$ to $p6$ were set to 1 and, for all mRNA species, $\mathrm{pp1_{mrna}}$ to $\mathrm{pp6_{mrna}}$ were set to 0. The observation period is given by $t = [0, 20]$.

References

Becker, V., Schilling, M., Bachmann, J., Baumann, U., Raue, A., Maiwald, T., Timmer, J., and Klingmuller, U. (2010). Covering a Broad Dynamic Range: Information Processing at the Erythropoietin Receptor. *Science*, **328**(5984), 1404–1408.

Brooks, S. P. (1998). Markov Chain Monte Carlo Method and Its Application. *Journal of the Royal Statistical Society. Series D (The Statistician)*, **47**(1), 69–100.

Fuguitt, R. E. and Hawkins, J. E. (1947). Rate of the Thermal Isomerization of α-Pinene in the Liquid Phase. *Journal of the American Chemical Society*, **69**(2), 319–322.

Gelman, A., Carlin, J. B., Stern, H. S., Dunson, D. B., Vehtari, A., and Rubin, D. B. (2013). *Bayesian Data Analysis*. Chapman and Hall/CRC, 3 edition edition.

Kyung, M., Gill, J., Ghosh, M., and Casella, G. (2010). Penalized regression, standard errors, and Bayesian lassos. *Bayesian Analysis*, **5**(2), 369–411.

Li, C., Donizelli, M., Rodriguez, N., Dharuri, H., Endler, L., Chelliah, V., Li, L., He, E., Henry, A., Stefan, M. I., Snoep, J. L., Hucka, M., Novère, N. L., and Laibe, C. (2010). BioModels Database: An enhanced, curated and annotated resource for published quantitative kinetic models. *BMC Systems Biology*, **4**(1), 1–14.

Meyer, P., Cokelaer, T., Chandran, D., Kim, K., Loh, P.-R., Tucker, G., Lipson, M., Berger, B., Kreutz, C., Raue, A., Steiert, B., Timmer, J., Bilal, E., DREAMVI-VII Parameter Estimation Consortium, Sauro, H. M., Stolovitzky, G., and Saez-Rodriguez, J. (2014). Network topology and parameter estimation: from experimental design methods to gene regulatory network kinetics using a community based approach. *BMC Systems Biology*, **8**(1), 13.

Milo, R. (2002). Network Motifs: Simple Building Blocks of Complex Networks. *Science*, **298**(5594), 824–827.

Ouyang, X., Huang, X., Jin, X., Chen, Z., Yang, P., Ge, H., Li, S., and Deng, X. W. (2014). Coordinated photomorphogenic UV-B signaling network captured by mathematical modeling. *Proceedings of the National Academy of Sciences*, **111**(31), 11539–11544. 00003.

Park, T. and Casella, G. (2008). The Bayesian Lasso. *Journal of the American Statistical Association*, **103**(482), 681–686.

Raue, A., Kreutz, C., Maiwald, T., Bachmann, J., Schilling, M., Klingmüller, U., and Timmer, J. (2009). Structural and practical identifiability analysis of partially observed dynamical models by exploiting the profile likelihood. *Bioinformatics*, **25**(15), 1923–1929. 00253.

Swameye, I., Müller, T. G., Timmer, J., Sandra, O., and Klingmüller, U. (2003). Identification of nucleocytoplasmic cycling as a remote sensor in cellular signaling by databased modeling. *Proceedings of the National Academy of Sciences*, **100**(3), 1028–1033.

Yi, T.-M., Kitano, H., and Simon, M. I. (2003). A quantitative characterization of the yeast heterotrimeric G protein cycle. *Proceedings of the National Academy of Sciences*, **100**(19), 10764–10769.

Zacher, B., Abnaof, K., Gade, S., Younesi, E., Tresch, A., and Fröhlich, H. (2012). Joint Bayesian inference of condition-specific miRNA and transcription factor activities from combined gene and microRNA expression data. *Bioinformatics*, **28**(13), 1714–1720.

Zou, H. and Hastie, T. (2005). Regularization and variable selection via the Elastic Net. *Journal of the Royal Statistical Society, Series B*, **67**, 301–320. 03777.
